# Artificial Intelligence in Coronary Artery Calcium Scoring

**DOI:** 10.3390/jcm13123453

**Published:** 2024-06-13

**Authors:** Afolasayo A. Aromiwura, Dinesh K. Kalra

**Affiliations:** 1 Department of Medicine, University of Louisville, Louisville, KY 40202, USA; 2 Division of Cardiology, Department of Medicine, University of Louisville, Louisville, KY 40202, USA

**Keywords:** artificial intelligence, computed tomography, cardiac, coronary artery calcium scoring

## Abstract

Cardiovascular disease (CVD), particularly coronary heart disease (CHD), is the leading cause of death in the US, with a high economic impact. Coronary artery calcium (CAC) is a known marker for CHD and a useful tool for estimating the risk of atherosclerotic cardiovascular disease (ASCVD). Although CACS is recommended for informing the decision to initiate statin therapy, the current standard requires a dedicated CT protocol, which is time-intensive and contributes to radiation exposure. Non-dedicated CT protocols can be taken advantage of to visualize calcium and reduce overall cost and radiation exposure; however, they mainly provide visual estimates of coronary calcium and have disadvantages such as motion artifacts. Artificial intelligence is a growing field involving software that independently performs human-level tasks, and is well suited for improving CACS efficiency and repurposing non-dedicated CT for calcium scoring. We present a review of the current studies on automated CACS across various CT protocols and discuss consideration points in clinical application and some barriers to implementation.

## 1. Introduction

Coronary heart disease (CHD) is the most common type of cardiovascular disease (CVD) in the United States, and it accounted for 696,937 deaths in 2020 [1]. In addition, approximately USD 407.3 billion in CVD-associated costs were incurred in the US between 2018 and 2019. Considering the fact that around 50% of CVD-associated deaths occur in asymptomatic individuals or patients without prior CVD diagnoses, atherosclerotic CVD (ASCVD) risk stratification and modification are essential aspects for ensuring CVD prevention and subsequent disease cost reduction, early diagnosis, and the reduction of mortality and major adverse cardiovascular events (MACE) [2]. Coronary artery calcium (CAC) is an established indicator of coronary artery disease (CAD) that is associated with disease burden and ASCVD risk and is quantified by ECG-gated non-contrast computed tomography (NCCT) [3,4]. CAC scoring (CACS) by ECG-gated NCCT is a total measure of calcium density and plaque attenuation, which may be used to inform the decision for therapy initiation [4]. The Agatston method for CACS estimates coronary artery calcium lesion scores, defined as the product of the total area of calcified plaque (in squared millimeters) and the peak calcium density weighing score [5]. Absolute CAC scores are then assigned to each coronary vessel based on the maximal Hounsfield units (HU) and may be associated with a visual score such that 0 = none, 1–10 = minimal, 11–100 = mild, 101–400 = moderate, and >400 = severe. Visual scores are subjective estimations of CAC based on visual analysis of non-ECG-gated scans [6].

Several society guidelines including the American College of Cardiology/American Heart Association (ACC/AHA), European Society of Cardiology, and National Lipid Association have recommended the use of CACS to guide preventive therapy initiation in specific populations [7]. The ACC/AHA recommends CACS in patients with intermediate risk and some with borderline risk and uncertainty regarding risk-based preventive therapy; statin initiation is recommended when CACS ≥ 100 [8]. Other risk stratification tools, such as the Multi-Ethnic Study of Atherosclerosis (MESA) risk score, a race-, age-, and sex-adjusted score that incorporates CACS with other known CAD risk factors, have been shown to improve the estimation of 10-year CVD risk. However, the extensive use of CACS is still limited by several factors including its lack of insurance coverage, the need for a dedicated ECG-gated NCCT protocol, and the need for specialized imaging lab personnel, particularly in high-volume imaging labs and institutions. Although the application of non-dedicated CTs (CTs acquired for other purposes besides coronary artery calcium scoring) to manual CACS and calcium visual estimation has been described by several authors to have good correlation to Agatston scoring, it is not the current standard of practice [9,10,11].

As artificial intelligence (AI) continues to play an increasing role in medicine and radiology, it offers unique advantages to CACS and CVD risk estimation [12,13,14]. Beyond improving the time-consuming, labor-intensive, and repetitive tasks of calcium segmentation and quantification, AI offers the possibility of acquiring quantitative CACS from non-dedicated routine chest CT protocols acquired for other purposes, including low-dose CT (LDCT), cardiac CT angiography (CCTA), and positron emission tomography (PET)/CT attenuation scans. Non-dedicated CT use also has the advantage of decreased summative radiation exposure. There are a few existing commercially available semiautomatic and automatic CACS software products, some of which were utilized in the studies discussed below (Table 1). Although semi-automatic tools are useful, they still require manual allocation of automatically segmented and detected calcium; therefore, they are user-dependent and time-consuming.

## 2. Deep Learning and Artificial Neural Networks

The multi-layered neural networks of deep learning (DL) algorithms learn from inputs of complex datasets and automatically produce outputs by recognizing intricate data relationships that might otherwise be missed at a human level. DL techniques are widespread and commonly applied in speech recognition, computer vision, and language processing. In recent years, DL has been increasingly applied across various cardiovascular imaging modalities for various tasks including image acquisition, reconstruction, segmentation, analysis, and prognosis [16]. In particular, convolutional neural networks (CNNs) are the most commonly used in cardiovascular imaging and automated CACS. CNNs comprise several specialized network layers that identify image-based features and can create feature maps that aid the prediction of the final model output [17].

## 3. Studies of CACS Automation

**Figure 1:**
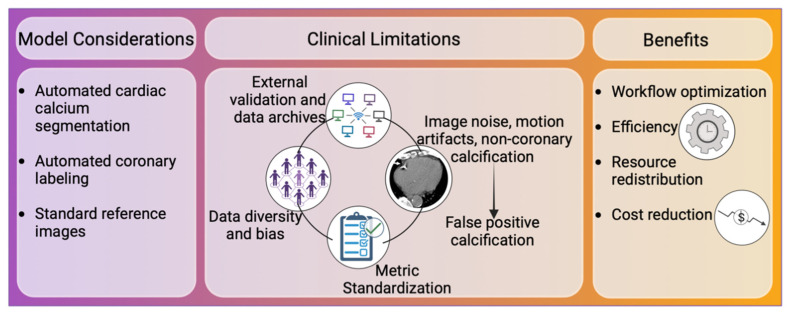
Overview of Artificial Intelligence in Coronary Artery Calcium Scoring. Automatic cardiac segmentation and coronary labeling are hallmarks of proposed models. Improvement of data diversity, data repositories, external validation, image artifacts, and metric standardization will enhance the proposed models. CACS automation optimizes workflow, reduces cost, and allows redistribution of resources in image post-processing labs.

A fully automated CACS software product should be able to perform coronary artery segmentation, coronary calcium analysis, and coronary artery labeling (Figure 1). Given the role of CACS as a risk stratification tool, the process of automatic CACS model development and testing should demonstrate excellent generalizability and ability to assess the risk of ASCVD across a diverse population. Models that are limited in dataset size and diversity tend to have overfitting and poor generalizability, a situation where they perform well with training data, but are unable to make accurate predictions with test data; these models underperform in the real world [17,18]. The training data typically form the majority of the entire dataset and are utilized for model development, while the test data are used to assess model performance after model training [17]. Some of the consequences of poor generalizability are reductions in accuracy and greater variability in model sensitivity and specificity when tested against external datasets. For instance, one CNN algorithm was developed for hepatic fibrosis categorization on ultrasound images and demonstrated an accuracy of 83.5% when tested against the internal dataset; however, the model accuracy reduced to 76.4% with external testing, reflecting the model’s inability to generate a reliable output when small changes were made to its input data such as a new or external testing dataset [19]. Therefore, the assessment of model generalizability through external validation is essential. The development of diverse multi-detector and multi-institutional data banks will be necessary for building clinically relevant AI risk assessment models.

Beyond the major task performances, the reduction of false positives and appropriate reference standards are necessary for reliable model development. Proposed models should have the ability to focus on target coronary anatomy to avoid misclassification of aortic and valvular calcifications as coronary calcifications [20,21,22]. Various approaches have been described for region-of-interest localization and segmentation including conventional manual segmentation methods, bounding boxes with manual lesion localization via coordinates, and automatic coronary extraction [20,23,24]. Additionally, though gated and non-gated NCCT CACS have been shown to have good agreement, they may differ in mean absolute scores, so manually derived gated CACS are preferred as a reference standard for comparing automated scores. However, in models trained on non-dedicated CT, reference manual scores from non-dedicated CT also allow the independent evaluation of model accuracy.

### 3.1. ECG-Gated and Non-Gated NCCT

**Figure 2:**
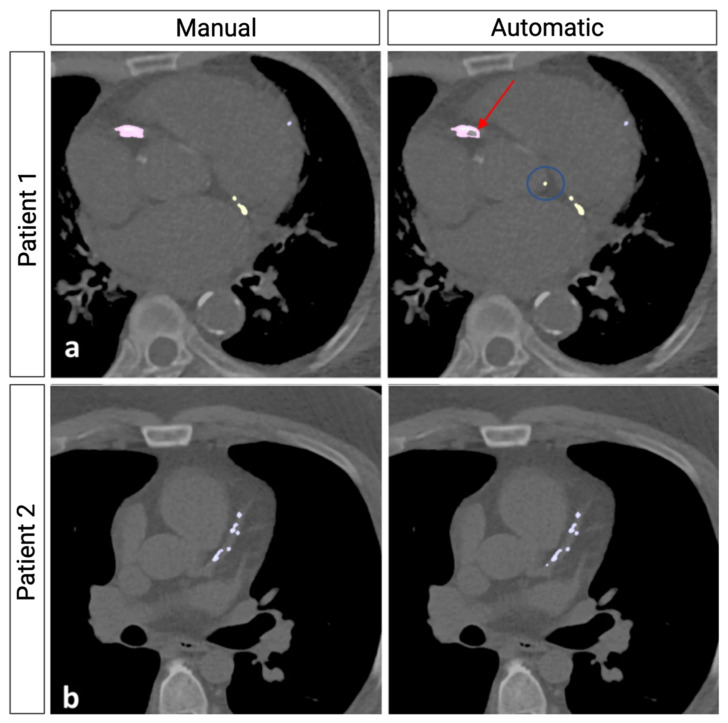
Comparison of Manual and Automatic CAC Identification. Image adapted from Eng et al. [25] Manual and automatic CACS comparison in two patients. The automatic model excludes calcifications in the descending aorta and some in the aortic root. (**a**) False negative calcium analysis by the automatic model in the right coronary artery (red arrow). False positive aortic root calcification is present (blue circle). (**b**) Comparison of automatic and manual coronary calcifications in another patient.

Over time, various approaches, model types, and datasets have been proposed for CACS automation with varying results (Table 2). One study, by Eng et al., proposed two CNN-based models for automatic scoring using gated coronary CT and non-gated chest CT, respectively (Figure 2) [25]. Automatic scores from both models demonstrated an almost-perfect correlation with manual scoring (Cohen’s kappa 0.89, *p* < 0.0001). The non-gated model also had very good sensitivity (71–94%) and positive predictive value (PPV) (88–100%) and the gated model had a faster average scoring time (3.5 ± 2.1 s vs. 261 s). Other researchers have proposed models trained on gated CT images and compared them to gated CT-derived reference manual scores. For instance, Ihdayhid et al. used a CNN-based model developed by Artrya Ltd. for automatic CACS in gated NCCT scans [22]. The model included separate CNNs for aortic and cardiac segmentation to distinguish aortic from coronary calcifications and decrease the false positive rate of the proposed model. Overall, the proposed model efficiently produced automatic scores that had excellent agreement with manual scoring (κ = 0.90 [95 CI, 0.88–0.9], *p* < 0.001).

While most studies used one or two CNN-based models, a multicenter, multi-scanner, and multivendor study trained and tested an ensemble of five 3D U-Net models [37]. The ensemble model trained on all data subsets achieved the best Cohen’s kappa (0.894), accuracy (85.7%), and intraclass correlation coefficient (ICC) (0.970) relative to single models and the ensemble model trained on partial data subsets. This study further highlighted the benefit of a large training dataset size on model performance. Two separate studies compared their suggested automatic tools with established semi-automatic software and had good outcomes. The first report compared a proposed automatic software product with a reference semi-automatic software product, syngo.via, and showed excellent correlation by Pearson’s coefficient (0.935) and ICC (0.996); furthermore, good risk category assignment was also achieved (κ = 0.919) [38]. Similarly, another study proposed a model based on an improved U-Net structure, known as U-Net++, and compared it to syngo.CT CaScoring, a semiautomatic software product [36]. The model was tested on two datasets, with and without clinically detected CAC. The model achieved excellent ICC (1.0) for Agatston, volume, and mass scores relative to semi-automated CACS and kappa for risk categorization.

A 2021 study by Xu et al. was unique in its comparison of automatic scoring from non-gated chest CTs of varying slice thicknesses, 1 mm and 3 mm [23]. Manual gated NCCT was used as a reference, and though both slice thicknesses had good ICC (1 mm = 0.9 [95% CI, 0.85–0.93] and 3 mm = 0.94 [95% CI, 0.92–0.96]), PPV (1 mm = 90% vs. 3 mm = 93%), accuracy (1 mm = 88% vs. 3 mm = 94%), and κ (1 mm = 0.72 and 3 mm = 0.82), automatic scores from the 3 mm scans demonstrated slightly better values overall. Although this study showed improved accuracy with thicker slice scans, further analyses are required to determine the optimal thickness for model accuracy and noise reduction considering that noise increases with thickness and contributes to calcium score misclassification.

A quality improvement project by Sandhu et al. derived automated CACS from previously acquired non-gated NCCT and showed 51% statin prescription for primary prevention at 6 months when primary care physicians were notified of CAC presence, versus 7% without notification [32]. The study also demonstrated a significant decrease in low-density lipoprotein and an increase in further non-invasive CAD testing in the notification arm. The study highlights the potential for improved preventative care with AI, non-dedicated CT, and results notification; further studies on its impact on MACE would be enlightening. A cardiovascular outcomes study utilized automated CAC derived from non-gated NCCT and determined that CAC ≥ 100 was associated with a higher risk of death, as well as a composite of death/MI/stroke and death/MI/stroke/revascularization, relative to CAC = 0 [33]. Furthermore, patients were being undertreated with statins.

### 3.2. PET/CT Attenuation Correction

Non-gated CT attenuation correction scans in PET imaging have also been used for automatic CACS. Pieszko et al. proposed a DL model for automated CACS from CT attenuation correction (CTAC) scans acquired with cardiac PET imaging [26]. There were no significant differences in net reclassification and the model showed a stepwise increase in MACE in the CACS groups. Similarly, standard CACS and DL-based CTAC scores had similar negative predictive values (NPV) for MACE at 85% and 83%, respectively.

One study used a U-Net-based model, AVIEW CAC by Coreline Soft, for automatic CACS in ungated CT scans from 100 patients who underwent 18-FDG PET/CT [27]. Patients in this study also underwent ECG-gated CT scans within 6 months of PET/CT that were manually scored and compared to the AI-based CACS from ungated CT. Though the model had almost perfect risk categorization κ, it underestimated the CAC category in 42% of cases and 44% had to be recategorized.

Another group presented a model for automatic CAC quantification in CCTA scans [24]. The volume of interest was first automatically determined by placing a “bounding box” around the heart for extracardiac calcification exclusion. Four ConvPairs were generated based on dimensionality (2.5D and 3D) and input patch size (15 or 25 voxels) and used for voxel classification; overall, the best-performing ensemble had a 71% sensitivity and 0.48 FP errors per scan. The model also had excellent ICC and an almost perfect κ relative to the reference, gated NCCT mass score.

### 3.3. Low-Dose Chest CT and Transthoracic Echocardiogram

LDCT has also been applied towards automatic CAC scoring with good outcomes. One study analyzed automatic CACS using AVIEW CAC by Coreline Soft in LDCT and gated CT across three institutions [28]. Compared to manual scores from both scan types, the LDCT scores were substantial to almost-perfect κ, while gated CT scores were almost perfect κ across all institutions. LDCT also had a higher false positive rate which may have been contributed by more noise and artifacts or poorer model performance due to lymph node calcifications. Another study assessed automated CACS, using AVIEW by Coreline, as a predictor of 12-yr all-cause mortality in LDCT and showed an incremental association between CACS and all-cause mortality [29].

Yu et al. used a commercially available automatic CACS software product, CACScore Doc by ShuKun technology, that was trained and tested on non-gated chest CT for CAC quantification [35]. Automatic software was compared to semiautomatic software, CaScoring, Syngo by Siemens, and manual scoring.

Interestingly, a study by Yuan et al. employed a video-based CNN model for binary CACS prediction from transthoracic echocardiograms (TTE) [30]. The model was able to discriminate zero CAC from high CAC (≥400 Agatston units) with very good AUC and F1 scores for zero and high CAC. Though binary CACS is less informative in guiding therapy initiation due to the loss of information on patients with CACS between >0 to <400, the study shows promise for a potential role for TTE in automated CACS.

### 3.4. Multiple CT Protocols

Using data from four large cohort studies, Zeleznik et al. trained a model for cardiac segmentation and CAC quantification using manually segmented ECG-gated CTs and tested the model with both gated and low-dose chest CTs [31]. There was excellent correlation between the automatic and manual calcium score groups; however, the AUC for event prediction between the two was not statistically significant. A CNN-based algorithm suggested by Van Velzen et al. was tested on a diverse set of CT protocols including standard ECG-gated CT, LDCT, CTAC, diagnostic chest CT, and radiation therapy planning CTs with excellent ICC for CAC volume and overall κ [34].

## 4. Towards Clinical Implementation

### 4.1. Workflow Optimization

Presently, several institutions are employing CACS automation algorithms in clinical settings for use in routine non-dedicated CTs and standard, gated NCCTs. AI is able to improve the workflow in 3D imaging post-processing labs, particularly at high-volume centers. Automatic CACS models can improve efficiency and resource utilization by decreasing time spent on segmentation and coronary annotation. Though manual double-checks will be required to prevent oversight, the combined time for automatic scoring and double-checking has been shown to be shorter than manual scoring. Consequently, radiologists can use less time overall by mainly verifying the automated results, and radiology technicians can be repurposed for other post-processing tasks. This will also aid in cost reduction in imaging labs. In addition, high inter-observer variability in lower-quality non-dedicated scans can also be reduced by AI.

### 4.2. Image Considerations

Overall, the problem of miscategorization and false positive calcifications persists in CACS automation and has been attributed to artifacts, noise, and non-coronary calcifications (aortic, valvular, and lymph node calcifications) [20,22]. Denoising, region-of-interest localization, and segmentation are potential areas of intervention to improve image quality and reduce false positive calcifications. Multiple studies have demonstrated the superiority of iterative reconstruction (IR) over filtered back projection (FBP) for noise reduction during image reconstruction [39,40]. One study showed decreased radiation dose, higher signal-to-noise ratio (SNR), and fewer false positive calcifications with low-dose iterative reconstruction by establishing noise thresholds; low-dose IR exhibited better performance than filtered back projection [41]. Furthermore, DL-based image reconstruction (DLIR) may be an even better alternative to IR and FBP. However, a study showed that while incremental DLIR was associated with a stepwise decrease in noise and an increase in SNR, there was a tendency for significant incremental decreases in CACS and CAC volume [42]. As such, further research should be conducted in applying noise reduction to DL reconstruction to identify the optimal noise reduction level through DLIR without compromising quantitative CACS.

### 4.3. External Validation and Data Diversity

Training dataset diversity and external validation are essential for AI algorithm generalizability. Studies have shown the importance of large databases in model training and demonstrated inferior ICC and Cohen’s kappa when a model is trained with a smaller dataset [36]. Poorly generalizable models perform well with training datasets and poorly with test sets that differ from their training sets, a phenomenon known as overfitting. As such, AI models need diverse training datasets to excel in realistic clinical settings and prevent the propagation of real-world biases [43]. This goal can be attained through the creation of large multicenter, multivendor, multi-detector, and multi-phenotype databases, achievable through cross-institutional collaboration. For instance, BunkerHill Health has an existing research consortium including several academic institutions to facilitate faster multicenter cooperation by aiding AI algorithm training and validation, clinical deployment, and addressing the legal implications of multicenter partnerships [44]. Large dataset repositories exist for other imaging modalities including CMR and SPECT. Current research endeavors in model development can contribute to data archives by publicizing their annotated datasets and permitting their collation by research consortiums.

### 4.4. Metrics Standardization

During study evaluation, metric selection is essential for appropriate criteria evaluation and optimization during model training. Cohen’s kappa, concordance index, and ICC scores are ideal for assessing the risk category allocation, accuracy, and reliability; as such, they are commonly used metrics in automated CACS [45,46]. The Proposed Requirements for Cardiovascular Imaging-Related Machine Learning Evaluation (PRIME) checklist provides a guideline for reporting model evaluation measures including Bland–Altmann plots, misclassification risk, accuracies, and inter-/intra-observer variability; however, specific and standard acceptable parameter thresholds are undefined [47]. For example, Cohen’s kappa ranges from 0–1.0 with six different subgroups, the best of which is between 0.81–1.00, and is indicative of almost-perfect risk categorization [48]. However, there is no standard minimum acceptable value for automated models. A minimum requirement of almost-perfect categorization would be acceptable for proposed models; nevertheless, the requirement needs to be defined for Cohen’s kappa and other metrics to give more meaning to each model’s clinical adequacy.

## 5. Conclusions

AI application in CACS has the potential to produce precise, efficient, and accurate scores from dedicated and non-dedicated CT. While most studies presented so far have produced fast and accurate automatic scores, the challenges of data homogeneity, inadequate dataset size, and calcium mis-categorization due to noise artifacts and aortic/valvular calcifications remain persistent. Although a certain degree of false positive rates is expected with imaging tests, it is essential to minimize this in the higher CAC risk groups (>100 AU) where results significantly impact medical decision-making, to avoid over-utilization of resources for primary prevention and further diagnostic testing. Furthermore, creating data consortiums will support large-scale studies, facilitate accurate model development, and aid clinical implementation; however, an equal representation of samples from various demographics is essential to prevent the proliferation of real-world biases and disparities through biased datasets and AI models.

## Figures and Tables

**Table 1:** Commercially available systems for automatic CACS.

Company	Software	Applications
General Electric Healthcare, IL, United States	CardIQ Suite	Coronary calcium segmentation and coronary artery labeling with total and per-region scoring.
Arytra Ltd., West Perth, Australia	DeepC architecture	Coronary artery calcium detection and plaque volume measurement.
Coreline Soft, Seoul, Korea	AVIEW CAC	Coronarartery segmentation, calcium analysis, and automatic report generation.
Nanox AI, Petah Tikva, Israel	HealthCCSng	Coronary calcium quantification and categorization.
Siemens Healthineers, PA, United State	AI-Rad Companion (Cardiovascular)	Coronary calcium and heart volume quantification in gated and non-gated NCCT. Does not perform Agatston scoring [15].

**Table 2:** Artificial intelligence in CACS.

Author	N	Test Image Type/Protocol	Reference Image	Results	Model
Eng et al., 2021 [25]	Gated CT model (retrospective 866 scans, prospective test 55 scans) Non-gated routine chest CT (341 training, Stanford total 215 scans [42 test], MESA total 232 scans [46 test], external validation 303 scans)	- Gated NCCT - Non-gated routine chest CT	- Gated NCCT manual - Non-gated manual NCCT	For CACS ≥ 100 in non-gated CT model: - Sensitivity = 71–94% - PPV = 88–100% For gated NCCT: - Cohen’s kappa = 0.89, *p* <0.0001 - AI processing time vs. manual scoring = 3.5 ± 2.1 s vs. 261 s	CNN
Ihdayhid et al., 2023 [22]	Training 2439 cardiac CT scans Validation 771 scans Test set 1849 cardiac CT scans	- ECG-gated NCCT scans	- ECG-gated NCCT	AI vs. Manual CACS: - Spearman’s r = 0.90 [95% CI, 0.89–0.91], *p* < 0.001 - ICC = 0.98 [95% CI, 0.98–0.99], *p* < 0.001 - Bland–Altman = 1.69 - κ = 0.90 [95 CI, 0.88–0.9], *p* < 0.001 - Analysis time = 13.1 ± 3.2 s/scan	DeepC Architecture (3D-CNN)
Pieszko et al., 2023 [26]	Training and internal testing 9543 (1827 gated CT and 7716 CTAC) External validation 4331 (2737 had ECG-gated NCCT)	- CT attenuation correction scans for AI - ECG-gated NCCT	- ECG-gated NCCT	- Automated scoring time = <6 s/scan - Net reclassification improvement = −0.02 [95% CI, −0.11–0.07] - NPV of DL CTAC = 83%	DL
Morf et al., 2022 [27]	Test set 100 patients	- non-gated NCCT for AI - ECG-gated CT	- ECG-gated NCCT	Per-patient AI CACS: - sensitivity = 85% - specificity = 90% Inter-score agreement = 0.88 [95% CI: 0.827, 0.918] κ = 0.9 Interscore agreement per-vessel = 0.716	AVIEW CAC (U-Net)
Wolterink et al., 2016 [24]	250 CCTA and 250 gated NCCT scans	- CCTA - ECG-gated NCCT	- Manual CCTA and ECG-gated NCCT	Pearson’s = 0.950 ICC = 0.944 CVD risk accuracy = 83% κ = 0.83 Sensitivity/FP = 0.72/0.48 Bland–Altmann = −0.2 (−38.7–38.3)	ConvPairs (CNN)
Suh et al., 2023 [28]	452 subjects (across 3 institutions)	- LDCT - ECG-gated NCCT	- Manual ECG-gated NCCT - Manual LDCT	Comparison of automatic and manual LDCT: κ = 0.972–0.918 Comparison of automatic and manual gated NCCT κ = 0.748–0.924	AVIEW CAC, Coreline Soft
Sabia et al., 2022 [29]	1129 subjects (Multicenter Italian Lung Disease Trial)	- LDCT	- Manual LDCT scoring	All-cause mortality CAC > 400: Hazard ratio = 5.75 [95% CI, 2.08–15.92]	AViEW, Coreline Soft (U-Net structure)
Yuan et al., 2023 [30]	2831 TTE videos paired with gated NCCT	- 32-frame TTEs in parasternal long-axis view	- Manual CACS by gated NCCT	AI TTE zero CACS vs. high CACS - AUC = 0.81 [95% CI, 0.74–0.88] vs. 0.74 [0.68–0.8] - F1 score = 0.95 vs. 0.74	CNN
Zeleznik at al., 2021 [31]	Test set 1857 ECG-gated CTs and LDCT NLST 14,959 patients FHS-CT2 663 PROMISE 4021 ROMICAT-II 441	- LD chest CT (NLST) - ECG-gated (FHS-CT2, PROMISE and ROMICAT-II)	- Manual CACS by LDCT and gated NCCT	Automatic = 1.938 s per scan κ = 0.70	U-Net
Xu at al., 2022 [22]	Training set 150 (group A—1 mm slice thickness) and 170 (group B—3 mm) chest CTs Test set 144 (1 mm) and 144 (3 mm) chest CTs External validation 344 paired scans	- ECG-gated NCCT - non-gated chest CT	- manual CACS by gated NCCT	Agreement between AI and gold standard manual CACS: - ICC Group A = 0.9 [95% CI, 0.85–0.93] - ICC Group B = 0.94 [95% CI, 0.92–0.96] Risk category classification: κ Group A = 0.72 κ Group B = 0.82 PPV, NPV, and accuracy: Group A = 90%, 83%, and 88% Group B = 93%, 98%, and 94%	U-Net
Sandhu et al., 2023 [32]	Test set 173 patients	Non-gated NCCT	Manual non-gated NCCT	Statin prescription in notification group vs. usual care group: 51.2% vs. 6.9%	DL
Peng et al., 2023 [33]	Test set 5678 adults	Non-gated NCCT	N/A	DL-CAC ≥ 100: - Mean 10 yrs ASCVD = 24% - 26% pts on statins DL-CAC ≥ 100 vs. DL-CAC = 0: - HR death = 1.51 [95% CI, 1.28–1.79] - HR death/MI/stroke = 1.57 [95% CI, 1.33–1.84] - HR death/MI/stroke/revascularization = 1.69 [95% CI, 1.45–1.98]	CNN
Van Velzen et al., 2020 [34]	Total 7240 ECG-gated standard CAC CT 902, CTAC 399, 1409 CTs radiation treatment planning (RadTherapy) 1409, 470 diagnostic Chest CTs, 2879 ECG-gated from JHS, 1181 NLST	LDCT ECG-gated CTAC	- Semi-automatic	Data for CAC CT, CTAC, diagnostic CT, and RadTherapy: ICC for CAC volume = 0.98, 0.97, 0.98, and 0.92, respectively Overall κ = 0.92 (95% CI, 0.91–0.93)	CNN
Yu et al., 2022 [35]	405 LDCT and 405 gated CT	LDCT Gated NCCT	LDCT and gated NCCT	Comparison with LDCT:	CACScoreDoc
Hong et al., 2022 [36]	1811 cases Training 754 Test 1 = 215 Test 2 = 744 Validation = 98	Gated NCCT	Semi-automated clinical software (syngo.CT CaScoring, Siemens)	Data for training dataset 1 ICC 1.00, *p* < 0.001 κ = 0.931 U-Net vs. U-Net++ - Dice = 0.54 vs. 0.86 - IoU = 0.54 vs. 0.84 - Precision = 0.54 vs. 0.88 Analysis time 50 ms per scan	U-Net++ (U-Net with immediate upsampling after downsampling)
Gogin et al., 2021 [37]	Test set 783 CT (SFR data challenge set) External validation 98 CTs (orCaScore challenge set)	Gated NCCT	- Manual CACS by gated NCCT	A five-ensemble model trained on all datasets: ICC = 0.970 κ = 0.894 Accuracy = 85.7%	CNN with 3D U-Net structure
Sandstedt et al., 2020 [38]	315 scans from SWEDEHEART registry	Gated NCCT	Semiautomatic and manual CACS	Correlation for CACS: Pearson’s = 0.935 ICC = 0.996 Bland–Altman = −8.2 (−115.1 to 98.2) κ = 0.919 Median analysis time: - semi-automatic = 59 s - automatic = 36 s	-

CTAC = Computed Tomography Attenuation Correction, FHS-CT2 = Framingham Heart Study, NLST = National Lung Screening Trial, PROMISE = Prospective Multicenter Imaging Study for Evaluation of Chest Pain, ROMICAT-II = Rule Out Myocardial Infarction using Computer Assisted Tomography II, JHS = Jackson Heart Study, LDCT = Low dose CT, NCCT = Non-contrast CT, ICC = Intraclass correlation.

## Abbreviations

ACC/AHA = American College of Cardiology/American Heart Association, AI = Artificial Intelligence, ASCVD = Atherosclerotic Cardiovascular Disease, CCTA = Cardiac Computed Tomography Angiography, CT = Computed Tomography, CTAC = Computed Tomography Attenuation Correction, CNN = Convolutional Neural Network, CAC = Coronary Artery Calcium, CACS = Coronary Artery Calcium Score, CAD = Coronary Artery Disease, CHD = Coronary Heart Disease, DL = Deep Learning, DLIR = Deep Learning Image Reconstruction, FBP = Filtered Back Projection, IR = Iterative Reconstruction, ICC = Intraclass Correlation Coefficient, LDCT = Low-Dose Computed Tomography, MACE = Major Adverse Cardiovascular Event, MESA = Multi-Ethnic Study of Atherosclerosis, NPV = Negative Predictive Value, NCCT = Non-Contrast Computed Tomography, PPV = Positive Predictive Value, PET = Positron Emission Tomography, PRIME = Proposed Requirements for Cardiovascular Imaging-Related Machine Learning Evaluation, SNR = Signal-to-Noise Ratio, TTE = Transthoracic Echocardiogram.
